# The Importance of Studying Infectious and Parasitic Diseases of Wild Animals in the Amazon Biome with a Focus on One Health

**DOI:** 10.3390/vetsci12020100

**Published:** 2025-02-01

**Authors:** Felipe Masiero Salvarani, Hanna Gabriela da Silva Oliveira, Letícia Yasmin Silva Correa, Aline Alessandra Lima Soares, Brenda Cabral Ferreira

**Affiliations:** Instituto de Medicina Veterinária, Universidade Federal do Pará, Castanhal 68740-970, PA, Brazil; hanna.oliveira@castanhal.ufpa.br (H.G.d.S.O.); leticia.correa@castanhal.ufpa.br (L.Y.S.C.); aline.soares@castanhal.ufpa.br (A.A.L.S.); brenda.ferreira@castanhal.ufpa.br (B.C.F.)

**Keywords:** zoonotic diseases, biodiversity conservation, environmental health, One Health, disease transmission, wildlife health

## Abstract

The Amazon Biome is home to an extraordinary diversity of wildlife, many of which are reservoirs or vectors for infectious and parasitic diseases that can impact not only the health of wild animals but also human and domestic animal populations. This narrative review highlights the critical importance of studying infectious and parasitic diseases in wild animals within the Amazon, particularly in the context of the One Health approach, which recognizes the interconnectedness of human, animal, and environmental health. This narrative review examines key pathogens, including viruses, bacteria, and parasites, that pose significant risks to wildlife conservation and public health. Through a synthesis of recent literature, this article emphasizes the need for comprehensive surveillance, research, and collaboration between the veterinary, medical, and environmental sectors. The results underscore the urgent necessity for an integrated response to emerging diseases, particularly as environmental changes and human activities increasingly disrupt ecosystems in the region. The conclusions advocate for the reinforcement of One Health initiatives in the Amazon Biome to ensure the protection of biodiversity and the prevention of zoonotic disease transmission to human populations.

## Simple Summary

Wild animals in the Amazon Biome are exposed to numerous infectious and parasitic diseases that can significantly impact their health, well-being, and the entire ecosystem. Studying these diseases is essential not only to safeguard wildlife welfare but also to understand their broader implications for human and domestic animal health, highlighting the interconnectedness of health and the importance of promoting animal welfare as a cornerstone of ecosystem sustainability. This approach is known as One Health, which recognizes that the health of people, animals, and the environment are closely linked. By investigating diseases in wild animals, scientists can identify early warning signs of potential outbreaks that may spread to humans or livestock. Understanding how diseases develop and spread in this unique tropical environment is critical for protecting biodiversity and preventing larger health crises. This research can lead to better strategies for disease prevention and control, which benefits wildlife conservation efforts, public health, and sustainable development in the Amazon region.

## 1. Introduction

The Amazon Biome, encompassing approximately 6.7 million square kilometers across nine South American countries, is one of the most biologically diverse regions on the planet. This ecosystem hosts a wide range of wildlife species, many of which are not only critical to the ecosystem’s function but also serve as reservoirs for infectious agents, including viruses, bacteria, fungi, and parasites [1,2,3,4]. The interconnection between wildlife, humans, and the environment in this biome underlines the need to study diseases from a One Health perspective, which promotes the collaborative efforts of multiple disciplines to achieve optimal health outcomes for people, animals, and the environment [5,6,7]. The Amazon Biome, home to an unparalleled diversity of wildlife, plays a crucial role in maintaining the global ecological balance. Its vast landscapes house a wide variety of species, many of which remain understudied in terms of their interactions with infectious and parasitic diseases. Given that wildlife often serves as reservoirs for numerous pathogens, the potential for zoonotic transmission—diseases that can pass from animals to humans—is particularly concerning. This highlights the importance of understanding how infectious and parasitic diseases manifest within the biome’s wild animal populations and the wider implications for both human and environmental health [1,2,3,4,8,9].

Infectious and parasitic diseases in wild animals can disrupt ecosystem dynamics, cause population declines, and pose direct threats to biodiversity. These diseases often go unnoticed until they have caused significant impacts, as wild animals are difficult to monitor comprehensively [10,11]. The dense and remote nature of the Amazon further complicates efforts to study disease prevalence and transmission pathways. Moreover, the encroachment of human activities, such as deforestation, mining, and agricultural expansion, has brought humans, livestock, and wild animals into closer contact, creating new opportunities for disease spillover. In this context, a One Health approach—integrating human, animal, and environmental health—is essential to address these interconnected challenges [12,13]. The One Health concept recognizes that the health of humans, animals, and ecosystems are deeply intertwined, particularly in regions like the Amazon, where environmental degradation can directly influence disease emergence. As wildlife populations are increasingly stressed by habitat loss, they become more vulnerable to infections, which can spread rapidly within and between species. Understanding the role of wild animals in the epidemiology of infectious and parasitic diseases within the Amazon Biome can help predict and prevent outbreaks that may affect human populations or domestic animals and mitigate the ecological consequences of these diseases [1,2,3,4,7,8,9].

The urgent need to study infectious and parasitic diseases in wild animals within the Amazon is underscored by the rapid pace of environmental changes occurring in the region. As climate change accelerates and human impacts intensify, new diseases are likely to emerge, and existing ones may shift their geographic range or increase in prevalence. Comprehensive studies that integrate ecological, veterinary, and public health perspectives are required to address this growing threat [14,15]. By focusing on the Amazon Biome, this narrative review aims to shed light on the significance of infectious and parasitic diseases in wild animals and the critical need for research within the framework of One Health. As the human footprint in the Amazon grows due to deforestation, urbanization, and resource extraction, the interaction between humans and wildlife intensifies. This increased contact elevates the risk of zoonotic disease transmission—diseases that jump from animals to humans [16,17]. Recent pandemics, such as COVID-19 [18], have underscored the importance of monitoring and mitigating zoonotic threats, many of which originate from wildlife reservoirs. This narrative review focuses on the importance of studying infectious and parasitic diseases in wild animals in the Amazon Biome, providing a comprehensive analysis of the existing literature and identifying areas where further research is needed to better understand and control these diseases within the One Health framework.

## 2. Methodology Used in the Narrative Review

The present study is a descriptive research project based on a narrative literature review, as defined by Grant and Booth [19]. This review approach was chosen due to the broad scope of the topic and the need to synthesize existing knowledge on infectious and parasitic diseases in wild animals from the Amazon Biome and their relevance to One Health. To ensure methodological rigor, the search strategy included the use of multiple electronic databases, including Periódicos Capes, PubMed, Scopus, ResearchGate, Scielo, Google Scholar, Academia.edu, BDTD, Redalyc, Science.gov, ERIC, ScienceDirect, SiBi, World Wide Science, PePSIC, and Scholarpedia. The search terms employed—either independently or in combination—were infectious and parasitic diseases, wild animals, Amazon Biome, One Health, zoonotic diseases, biodiversity conservation, environmental health, disease transmission, and wildlife health. The inclusion criteria for selecting publications were as follows: articles published in peer-reviewed journals or credible scientific sources that addressed the specified search terms, provided relevant data or insights about wild animals in the Amazon Biome, and explored connections to One Health or zoonotic disease transmission. Exclusion criteria involved publications that were not directly related to the Amazon Biome or those with insufficient focus on infectious and parasitic diseases in wildlife. To assess the quality and relevance of the sources, all included publications underwent an evaluation based on their methodological soundness, sample size, and relevance to the scope of the review. A total of 86 unique publications were identified, with an overlap rate of 87% across the databases consulted. Given the limited number of references available on this topic, all retrieved publications were incorporated into the present review to ensure a comprehensive analysis. This transparent methodology not only supports the reproducibility of this review but also ensures that its findings represent a robust synthesis of the existing literature on this critical subject.

## 3. Infectious Diseases in Wild Animals of the Amazon Biome

### 3.1. Bacterial Diseases

The Amazon Biome, with its rich biodiversity, is host to a wide range of bacterial pathogens that can affect wild animals and have significant ecological, veterinary, and public health implications. These bacterial infections are not only a concern for wildlife health but also pose risks for zoonotic transmission, impacting humans and domestic animals. The study of bacterial diseases in wild animals of the Amazon is still in its infancy, and much remains unknown about the prevalence, transmission dynamics, and ecological consequences of these infections. However, the research to date highlights the pressing need for a more comprehensive understanding of bacterial pathogens circulating in this unique biome [20,21].

One of the most significant bacterial diseases in the Amazon Biome is leptospirosis, caused by *Leptospira* spp. This zoonotic bacterium affects a variety of wildlife species, including mammals such as rodents, marsupials, and primates, which act as reservoirs. Leptospirosis is transmitted primarily through the urine of infected animals, contaminating water sources that are then consumed by other animals or humans. In the Amazon, where water plays a central role in the ecosystem, the potential for leptospirosis transmission is heightened. The interaction between wildlife, domestic animals, and human populations, particularly in areas where deforestation and land use changes increase water contamination, creates an ideal environment for the spread of this disease. Studies have suggested that the prevalence of leptospirosis in wild animal populations may be underreported, calling for more targeted research and surveillance in the region [22,23,24,25].

Brucellosis is another bacterial disease of concern in the Amazon Biome, caused by *Brucella* spp. This disease affects various wildlife species, including ungulates, carnivores, and marine mammals, such as manatees and river dolphins. Brucellosis is a zoonotic infection that can have severe reproductive consequences for animals, including abortions, infertility, and reduced population viability. In the Amazon, brucellosis has been detected in several species of wild mammals, raising concerns about its impact on wildlife populations and its potential to spread to domestic animals and humans. The close proximity of livestock to wildlife in certain areas, coupled with the lack of veterinary infrastructure, increases the risk of cross-species transmission of *Brucella*. Monitoring and managing brucellosis in the Amazon are critical, as it poses a dual threat to conservation efforts and public health [22,25,26,27,28].

Another notable bacterial pathogen present in the Amazon is tuberculosis, caused by *Mycobacterium* spp. Tuberculosis has been reported in various wild animals, including primates, deer, and carnivores. In some cases, wild animals may serve as reservoirs for *Mycobacterium bovis*, which can infect domestic cattle and humans, particularly in rural areas where human–animal interactions are common. Tuberculosis is of particular concern in primate populations, where it can cause severe respiratory illness and contribute to population declines. Given the ecological importance of primates in the Amazon as seed dispersers and key components of the food web, the impact of tuberculosis on these species could have cascading effects on the ecosystem. Additionally, the potential for zoonotic transmission makes tuberculosis a priority for One Health approaches in the region [29,30,31].

Melioidosis, caused by *Burkholderia pseudomallei*, is a lesser known but emerging bacterial disease in the Amazon Biome. This soil-dwelling bacterium can infect a wide range of animals, including mammals, birds, and reptiles, and is capable of causing severe, often fatal, infections. In recent years, melioidosis has gained attention due to its increasing incidence in tropical regions, including parts of the Amazon. The bacterium can be transmitted through direct contact with contaminated soil or water, making wildlife populations in the Amazon particularly vulnerable. Given the environmental conditions of the Amazon, with its warm and humid climate, melioidosis may pose a growing threat to wild animals, especially as environmental disturbances increase soil and water contamination. Research into the epidemiology of melioidosis in the Amazon is still limited, but it is a disease that warrants further investigation due to its zoonotic potential and the risk it poses to wildlife [32,33,34].

In summary, bacterial diseases in the wild animals of the Amazon Biome represent a complex and understudied area of research. Pathogens like *Leptospira*, *Brucella*, *Mycobacterium*, and *Burkholderia pseudomallei* pose significant risks to wildlife populations and have the potential for zoonotic transmission, making them important targets for One Health initiatives. Future research must focus on improving surveillance, understanding transmission dynamics, and assessing the ecological and health impacts of these bacterial pathogens within the Amazon Biome [22,25,29,34].

Bacterial diseases such as leptospirosis, brucellosis, and tuberculosis have well-documented transmission cycles globally but remain understudied in the Amazon Biome. Leptospirosis, caused by *Leptospira* spp., is transmitted through the urine of infected animals, often rodents, which contaminates water sources, facilitating zoonotic and horizontal transmission to wildlife, livestock, and humans. In regions like Southeast Asia, rodents are key reservoirs, and similar dynamics are suspected in the Amazon, although the role of wildlife species such as marsupials and primates are poorly understood [23,24]. Brucellosis, caused by *Brucella* spp., impacts ungulates and carnivores and can cause reproductive issues such as infertility and abortion. In the Amazon, contact between wildlife and livestock due to habitat fragmentation likely increases cross-species transmission [26]. Similarly, tuberculosis, primarily caused by *Mycobacterium bovis* and *Mycobacterium tuberculosis*, infects a range of wildlife, including primates, which act as reservoirs and amplify the risk of human and livestock infections in shared habitats [29,31]. These diseases underscore significant gaps in the surveillance and the need for tailored One Health strategies in the Amazon.

### 3.2. Viral Diseases

**Figure 1:**
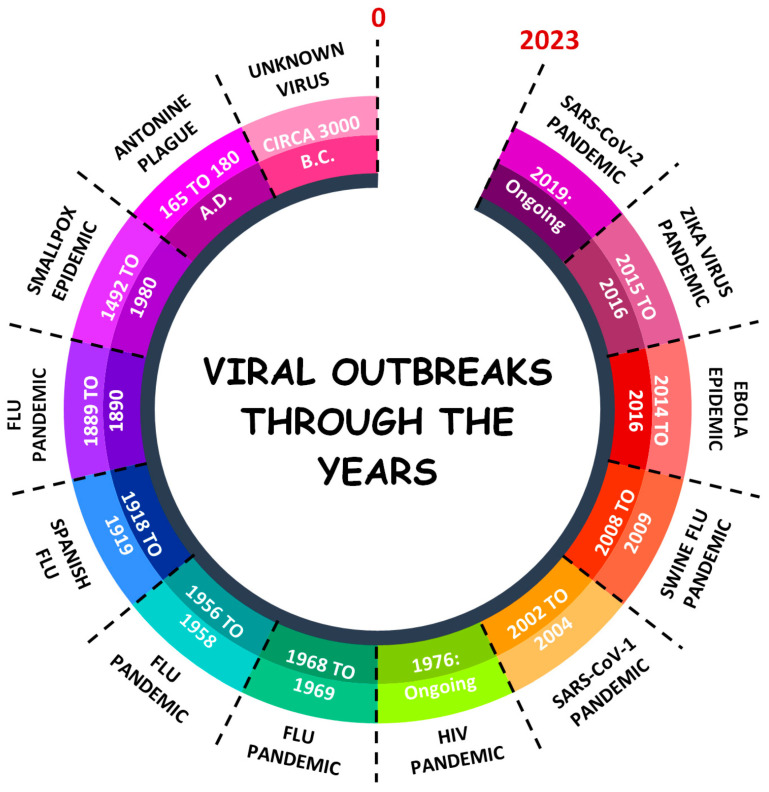
Ref. [35]: Timeline of history’s most notable viral pandemics and epidemics. Most major pandemics were attributed to mutated influenza viruses (H1N1, H2N2, and H3N2) that were thought to have originated in animal reservoirs and which subsequently spread to humans. The 2003 SARS-CoV-1 pandemic is regarded as the first pandemic of the 21st century and, similar to SARS-CoV-2, likely emerged from bats.

Viral diseases (Figure 1) present a significant threat to the wildlife of the Amazon Biome, which is home to numerous species that can act as reservoirs or vectors for emerging viruses. These viruses can have devastating effects on wild animal populations, affecting their health, behavior, and reproductive success. Moreover, given the close ecological interactions between species in the Amazon, viral outbreaks can spread rapidly across different animal populations, contributing to the transmission of pathogens to domestic animals and humans. Among the most concerning viral diseases in this context are those caused by zoonotic pathogens, which pose a direct threat to public health due to their potential to jump from animals to humans [35,36,37,38].

A key example of viral diseases with zoonotic potential in the Amazon is rabies, particularly in bat populations. Bats are known reservoirs for the rabies virus, and in many parts of the Amazon, cases of rabies transmission from bats to humans and livestock have been reported. Bats (*Desmodus rotundus*) are notorious vectors, feeding on livestock and occasionally transmitting the virus. Rabies outbreaks can have severe economic consequences, as livestock deaths from rabies reduce income for local farmers. In human populations, untreated rabies is almost invariably fatal, making its presence in wild animal populations a critical public health concern. Surveillance and control measures for rabies in bat populations, particularly in the context of One Health, are crucial for preventing spillover into human and domestic animal populations [39,40,41].

Another significant viral disease is West Nile virus (WNV), which has been documented in birds and mosquitoes in the Amazon. Birds act as amplifying hosts, and mosquitoes serve as vectors, transmitting the virus to other species, including humans and horses. While WNV is more commonly associated with outbreaks in North America, the Amazon region’s rich avian diversity and abundant mosquito populations make it a potential hotspot for viral circulation. Continuous deforestation and changes in land use can alter mosquito breeding grounds, increasing the risk of WNV transmission. This disease exemplifies the interconnectedness of environmental health, animal health, and human health and highlights the need for integrated monitoring systems to detect viral emergence and prevent outbreaks [42,43,44,45].

Additionally, emerging viral pathogens such as hantaviruses, which are transmitted by rodents, have been increasingly reported in the Amazon. Hantavirus pulmonary syndrome (HPS) is a severe respiratory disease in humans, and rodent populations in the region act as natural reservoirs for the virus. Infected rodents can transmit the virus to humans through contact with their saliva, urine, or feces. The encroachment of human populations into previously undisturbed areas of the Amazon, where these rodent species thrive, increases the risk of hantavirus outbreaks. Studies on the ecological dynamics of rodent populations and the impact of habitat disturbance on viral transmission patterns are crucial for understanding how environmental changes influence the emergence of zoonotic diseases like HPS [46,47,48].

Furthermore, the Amazon Biome’s dense tropical forest and warm, humid climate create ideal conditions for the persistence and spread of arboviruses, such as dengue, yellow fever, Zika, and Chikungunya viruses. These viruses have been detected in both wild animals and human populations in the region, highlighting their zoonotic potential and the interconnectedness of human, animal, and environmental health. In particular, the Chikungunya virus, primarily transmitted by *Aedes* mosquitoes, has shown the ability to establish sylvatic cycles involving non-human primates and other wildlife species, posing additional challenges to disease surveillance and control efforts. Understanding the role of wild animals as potential reservoirs for these arboviruses is essential for mitigating their impacts on public and veterinary health within a One Health framework. Non-human primates serve as important reservoirs for yellow fever and other arboviruses, and outbreaks in these populations can often precede human cases, serving as a warning signal for public health officials. The interplay between wild animal reservoirs, mosquito vectors, and human populations underscores the need for continuous surveillance of arboviral diseases within the Amazon [5,35,37,38,49,50,51,52,53].

Viral diseases in the Amazon are heavily influenced by interactions between reservoirs, vectors, and environmental conditions. Rabies, for instance, is predominantly maintained in bat populations (*Desmodus rotundus*), with transmission occurring through bites to livestock and humans, particularly in areas with intensified land use changes [39,40,41]. Similarly, arboviruses such as dengue, yellow fever, and Zika rely on mosquito vectors like *Aedes aegypti* and *Haemagogus* spp., with non-human primates serving as significant reservoirs for yellow fever. These cycles are well characterized globally but poorly defined in Amazonian ecosystems, where environmental disturbances alter vector breeding grounds and reservoir dynamics [42,43,49,50,51]. Hantaviruses, carried by rodent reservoirs, represent another threat, with transmission occurring through exposure to rodent excreta. Although outbreaks have been reported in Amazonian regions, the precise role of the local rodent species remains unclear [46,47,48]. Understanding these viral cycles is critical for mitigating spillover events that could lead to regional outbreaks or global pandemics.

### 3.3. Fungal Diseases

Fungal diseases are a significant yet often overlooked threat to wildlife health in the Amazon Biome. These pathogens can infect a wide range of animal species, from amphibians to mammals, and have the potential to cause severe morbidity and mortality. Unlike viral or bacterial infections, fungal diseases frequently develop slowly, making detection and management more difficult. In the Amazon, where biodiversity is immense, fungal pathogens can exploit the complex ecosystem, thriving in the region’s humid, warm conditions. This poses unique challenges for wildlife conservation and disease management, as the fungal life cycle and infection mechanisms can be difficult to interrupt without disrupting the broader ecological balance [54,55,56].

One of the most studied fungal diseases affecting wild animals in the Amazon is chytridiomycosis, caused by *Batrachochytrium dendrobatidis* (BD). This fungal pathogen has decimated amphibian populations across the world, and the Amazon is no exception. Amphibians, being key ecological indicators, are severely affected by chytridiomycosis, with species exhibiting mass mortality and population crashes in infected areas. The high humidity and temperature of the Amazon create an ideal environment for BD proliferation, making it an ongoing concern for amphibian conservation. The loss of amphibian biodiversity due to fungal infections like BD also has cascading effects on ecosystem health, as amphibians play critical roles in controlling insect populations and serving as prey for a variety of predators [57,58,59].

Fungal pathogens like *Histoplasma capsulatum* and *Aspergillus* species also pose threats to wild mammals, birds, and reptiles in the Amazon. *Histoplasma capsulatum*, the agent of histoplasmosis, is a soil-borne fungus that thrives in areas with high concentrations of bird or bat guano, which are abundant in the Amazon due to the large populations of these species. While histoplasmosis is primarily a zoonotic disease affecting humans, its impact on wildlife is still being explored. Birds and bats that harbor the fungus can spread the spores through their droppings, potentially infecting other animals that come into contact with contaminated environments. *Aspergillus* species, on the other hand, are opportunistic pathogens that can cause respiratory infections, particularly in birds. Aspergillosis can be lethal, especially in birds with weakened immune systems or those exposed to environmental stressors such as deforestation or pollution [60,61,62].

Fungal diseases in wild animals of the Amazon are not only a direct threat to the animals themselves but also serve as a potential reservoir for zoonotic transmission to humans. The porous boundaries between wildlife habitats and human settlements in the Amazon increase the risk of fungal pathogen spillover. As humans encroach further into forested areas for agriculture or logging, the likelihood of contact with infected wildlife or contaminated environments rises. In this context, the One Health framework becomes particularly relevant. For example, outbreaks of histoplasmosis in humans have been traced to deforestation and cave exploration, where people unknowingly disturb fungal spores in areas heavily populated by bats. Addressing fungal infections in wildlife through ecosystem health approaches is essential to mitigate both wildlife and human health risks [60,61,62].

Lastly, the changing environmental conditions in the Amazon, driven by climate change and human activity, may alter the prevalence and distribution of fungal pathogens. For instance, global warming and habitat fragmentation could create new ecological niches for fungi, expanding their range into areas where they were previously absent. Wild animals already stressed by habitat loss, pollution, and food scarcity may become more susceptible to fungal infections, which can act as opportunistic diseases in immunocompromised individuals. Research on the dynamics of fungal pathogens in wild animals of the Amazon is still limited, and much remains unknown about their long-term impact on biodiversity and ecosystem health. Future studies are needed to understand the full scope of fungal diseases and to develop strategies for monitoring and managing these infections in the context of the Amazon Biome’s unique challenges [54,55,56].

Fungal diseases in the Amazon, such as chytridiomycosis and histoplasmosis, highlight the interplay between environmental factors and host–pathogen interactions. Chytridiomycosis, caused by *Batrachochytrium dendrobatidis*, has decimated amphibian populations globally, and the Amazon’s humid climate provides an ideal environment for fungal proliferation. Amphibians serve as both hosts and reservoirs, with infection cycles involving environmental spores [54,56,57,59]. Similarly, *Histoplasma capsulatum*, a soil-dwelling fungus enriched by bat and bird guano, infects wildlife and potentially humans, though the zoonotic dynamics in Amazonian settings remain understudied [60,61,62]. Opportunistic fungi like *Aspergillus* spp. also pose threats to immunocompromised wildlife, particularly avian species exposed to deforestation-related stressors [54,55]. Despite the known global impacts of these fungal pathogens, limited Amazon-specific data impede conservation and health management efforts.

### 3.4. Parasitic Diseases

Parasitic diseases are a significant concern for wildlife in the Amazon Biome due to the diverse array of parasites that thrive in the region’s complex ecosystems. These parasites include protozoa, helminths (such as nematodes and trematodes), and ectoparasites like ticks and fleas, which can have profound impacts on their hosts. Wild animals in the Amazon, from mammals to birds, reptiles, and amphibians, are constantly exposed to these parasitic organisms. The effects of parasitic infections can vary widely, ranging from subclinical conditions to severe illness, which can reduce the fitness, reproductive success, and survival of affected animals. The dynamic and biodiverse nature of the Amazon promotes a wide range of host–parasite interactions, yet many remain poorly studied [63,64,65].

One of the most concerning parasitic diseases in the Amazon’s wildlife is caused by *Trypanosoma* spp., a protozoan parasite responsible for trypanosomiasis. Species such as *Trypanosoma evansi* and *Trypanosoma vivax* are known to affect both wild and domestic animals, with some strains capable of spilling over into human populations. In particular, *Trypanosoma cruzi*, the causative agent of Chagas disease, poses a zoonotic threat, as its reservoirs include wild mammals such as armadillos, opossums, and various rodent species. This parasite, *Trypanosoma cruzi*, is transmitted by triatomine bugs such as *Rhodnius prolixus*, *Triatoma maculata*, and *Panstrongylus geniculatus*, which thrive in the humid and dense forest environments of the Amazon. The close proximity of humans to wildlife in many parts of the Amazon increases the risk of transmission, emphasizing the importance of surveillance and control efforts [66,67,68,69].

Helminth infections are also widespread among wild animals in the Amazon. Nematodes such as *Strongyloides* spp., *Toxocara* spp., and *Trichuris* spp. infect a variety of mammalian hosts, including primates, carnivores, and ungulates. These parasites can cause gastrointestinal diseases, respiratory distress, and even systemic infections, depending on the species and parasitic load. In addition to nematodes, trematodes like *Fasciola hepatica* have been reported in wild animals, especially those that share aquatic habitats with domestic livestock. These helminth infections often remain underdiagnosed in wildlife, as most studies focus on domestic animals or humans, leaving critical gaps in understanding their epidemiology and impact on biodiversity [13,70,71,72].

Ectoparasites, including ticks, fleas, and mites, play a key role in the transmission of vector-borne parasitic diseases in the Amazon Biome. Ticks, for example, are vectors for a range of protozoan parasites such as *Babesia* spp., which can infect mammals like jaguars, sloths, and deer. Similarly, various arthropods, particularly sandflies (genus Lutzomyia in the New World and *Phlebotomus* in the Old World), are the primary vectors of *Leishmania* spp., the causative agents of leishmaniasis. This disease affects a wide range of hosts, including wild and domestic animals, as well as humans, highlighting its significance within the One Health framework. Although fleas and mites have been occasionally implicated in the mechanical transmission of *Leishmania* spp., their role as vectors remains limited and less significant compared to sandflies, which are responsible for the biological transmission of the parasite. This distinction emphasizes the importance of targeting sandflies in control and surveillance strategies to reduce the spread of leishmaniasis in the Amazon Biome. Leishmaniasis is a particularly pressing issue, as it is endemic to many parts of the Amazon, and wildlife species serve as reservoirs for the disease. The ability of ectoparasites to thrive in tropical climates, coupled with increasing habitat disturbance, raises concerns about the spread of parasitic diseases through wildlife populations and into nearby human communities [64,65,73,74].

The presence of parasitic diseases in wild animals of the Amazon Biome also has ecological consequences. Infected animals can suffer from malnutrition, reduced reproductive success, and even death, leading to shifts in population dynamics and ecosystem structures. In addition, the stress of parasitic infections can weaken animals’ immune systems, making them more susceptible to other infectious diseases. Parasites, therefore, contribute to the intricate web of health challenges faced by wild animals in the Amazon. Studying these parasitic diseases is not only important for understanding wildlife health but also for protecting the integrity of ecosystems and safeguarding the health of humans who share these environments [75,76,77].

Parasitic diseases in the Amazon are driven by complex host–parasite–vector interactions. *Trypanosoma* spp., including *T. cruzi* and *T. evansi*, involve wild mammals as reservoirs and triatomine bugs as vectors, transmitting Chagas disease and trypanosomiasis, respectively. While global data highlight armadillos, opossums, and rodents as primary reservoirs, Amazon-specific cycles remain underexplored [66,67,68,69]. Helminth infections, such as those caused by *Toxocara* spp. and *Fasciola hepatica*, affect wildlife and livestock sharing aquatic environments, with trematodes using snails as intermediate hosts. These dynamics are well documented elsewhere but insufficiently studied in Amazonian systems [70,71]. Ectoparasites like ticks and sandflies transmit diseases such as babesiosis and leishmaniasis, with the latter involving wild mammals as reservoirs. Habitat disturbances amplify the role of vectors and hosts, intensifying zoonotic risks [73,74,75]. Addressing these parasitic cycles in the Amazon is vital to curbing their ecological and public health impacts.

## 4. The One Health Approach: A Necessary Framework

One Health is an integrated approach that recognizes the interdependence of human, animal, and environmental health. In the Amazon Biome, this approach is particularly crucial given the region’s ecological complexity and the frequent overlap between human and wildlife habitats. Human activities such as deforestation, mining, agriculture, urban expansion, and infrastructure development play a significant role in degrading ecosystems. These disruptions not only threaten biodiversity but also alter disease dynamics by forcing wildlife into closer proximity with human populations, thereby increasing the risk of zoonotic disease transmission [78,79,80].

### 4.1. Ecosystem Changes and Disease Emergence

Ecosystem changes in the Amazon Biome, driven primarily by human activities, are increasingly recognized as significant factors in the emergence and spread of infectious and parasitic diseases. Deforestation, urbanization, agricultural expansion, and climate change are reshaping the landscape, altering wildlife habitats, and pushing wild animals into closer proximity with humans and domestic animals. These alterations not only disrupt the delicate balance of ecosystems but also create ideal conditions for the emergence and transmission of zoonotic diseases. The Amazon’s vast biodiversity, combined with these environmental stressors, presents a unique opportunity for pathogens to circulate, adapt, and spill over into new hosts, including humans and livestock [2,13,16,81,82,83].

Deforestation, in particular, has been linked to increased rates of disease emergence in tropical ecosystems. As forests are cleared, wildlife species lose their natural habitats, which forces them into fragmented areas or closer to human settlements. This increased interaction between wildlife and humans facilitates the transmission of infectious agents. For example, studies have shown that deforestation is associated with the rise of vector-borne diseases, such as malaria, as the loss of tree cover creates ideal breeding grounds for disease-carrying mosquitoes. Furthermore, the displacement of wildlife can lead to changes in predator–prey dynamics, resulting in population explosions of certain species, like rodents, which are known reservoirs for various zoonotic pathogens [42,44,76,81,82,83].

In addition to deforestation, climate change is a significant driver of disease emergence in the Amazon. As temperatures rise and rainfall patterns shift, the distribution of wildlife species and their associated pathogens is also altered. Warmer climates can expand the range of vectors such as ticks, mosquitoes, and flies, which transmit diseases like leishmaniasis, dengue, and Zika virus. Moreover, the stress induced by climate change on wildlife populations can weaken their immune systems, making them more susceptible to infections and increasing the likelihood of pathogen transmission within and between species. The increasing frequency of extreme weather events, such as floods and droughts, also disrupts ecosystems and can lead to the spread of waterborne diseases, such as leptospirosis and cholera, particularly in areas with poor sanitation [2,49,73,84,85].

Agricultural expansion and the intensification of livestock farming in the Amazon are additional contributors to the emergence of infectious diseases. The conversion of forested areas into pastures and croplands not only reduces biodiversity but also brings domestic animals into closer contact with wildlife. Livestock can serve as both reservoirs and amplifiers of zoonotic diseases, allowing pathogens to jump between species and potentially evolve into forms that are more transmissible or virulent in humans. The Amazon’s cattle industry, for example, has been linked to the spread of bovine tuberculosis, brucellosis, and foot-and-mouth disease. Additionally, the use of antibiotics and other veterinary drugs in livestock farming can contribute to the development of antimicrobial resistance (AMR), a growing global concern that poses a significant threat to both human and animal health. The improper use or overuse of these drugs can lead to the selection of resistant pathogens, which may persist in the environment and spread through various ecological pathways, including water systems, soil, and wildlife. In the Amazon Biome, where biodiversity is exceptionally high and ecosystems are fragile, the environmental impact of veterinary drug residues is particularly concerning. These residues can disrupt microbial communities, alter natural pathogen dynamics, and potentially affect non-target species, including wild animals that interact with contaminated environments. Such interactions can amplify the risk of zoonotic disease transmission and further complicate efforts to maintain ecosystem balance and One Health objectives. Addressing these risks requires a multidisciplinary approach, promoting sustainable practices, and ensuring responsible drug use to safeguard both environmental health and biodiversity [2,13,42,84,85].

Another critical factor in the emergence of diseases in the Amazon Biome is the hunting and consumption of wildlife, commonly referred to as bushmeat. As traditional livelihoods are disrupted by environmental changes and economic pressures, more people turn to hunting wild animals for food or income. This practice brings humans into direct contact with a variety of species that can carry dangerous pathogens, such as primates, rodents, and bats. The handling, butchering, and consumption of these animals create multiple opportunities for zoonotic pathogens to cross the species barrier, potentially leading to outbreaks of diseases such as Ebola, hantavirus, and coronaviruses. The COVID-19 pandemic has underscored the global risks associated with wildlife consumption and the importance of regulating and monitoring such practices to prevent future pandemics [5,18,22,35,37,46,47,85].

**Figure 2:**
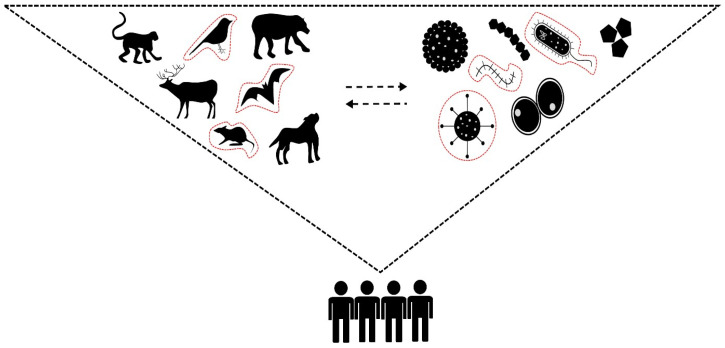
Ref. [35]: Alternative biodiversity models linking host and pathogen dynamics in the re-emergence of zoonotic diseases.

In light of these complex and interrelated factors, the One Health approach provides a necessary framework for understanding and mitigating the risks associated with ecosystem changes and disease emergence in the Amazon Biome. This holistic approach recognizes the interconnectedness of human, animal, and environmental health and calls for collaboration across disciplines to address the root causes of disease emergence (Figure 2). By integrating expertise from ecology, veterinary medicine, public health, and environmental science, the One Health approach aims to develop sustainable strategies for managing ecosystems, conserving biodiversity, and preventing the emergence of infectious diseases [35,41,79,85].

### 4.2. Interdisciplinary Collaboration

Interdisciplinary collaboration is a cornerstone of the One Health approach, essential for addressing the complex interplay between human, animal, and environmental health. The multifaceted nature of diseases, particularly those that emerge at the interface of wildlife, domestic animals, and humans, requires expertise across diverse fields. Veterinarians, medical doctors, epidemiologists, ecologists, microbiologists, public health experts, and environmental scientists must work together to comprehensively understand how diseases develop, spread, and impact various populations. However, to achieve the desired impact, it is also essential to include the participation of professionals in the fields of education and social sciences. These professionals play a critical role in bridging the gap between scientific findings and community engagement, ensuring that knowledge is effectively translated into practices and policies that resonate with local populations. In the context of the Amazon Biome, where ecosystems are exceptionally intricate and the risks of zoonotic spillover are heightened, such interdisciplinary collaboration becomes even more vital. By integrating expertise from diverse fields, a holistic approach can be achieved to mitigate the impacts of infectious and parasitic diseases, promote biodiversity conservation, and safeguard the health of both humans and wildlife under the One Health framework [7,41,42,79].

One of the primary benefits of interdisciplinary collaboration within the One Health framework is the ability to integrate knowledge across scales, from molecular- to ecosystem-level analyses. For example, a virologist may identify a novel pathogen in wild animal populations, but without the expertise of an ecologist or veterinarian, it would be difficult to determine the epidemiological significance of that pathogen. Similarly, public health experts can use this combined data to predict potential risks to human populations living in proximity to wildlife or those who rely on these animals for subsistence. The capacity to pool resources and expertise across disciplines enables a more holistic understanding of infectious and parasitic diseases, their reservoirs, and the factors that contribute to their emergence in wild animal populations [5,78,79].

Interdisciplinary collaboration also facilitates the development of innovative strategies for disease surveillance and control. In the Amazon Biome, traditional disease monitoring methods may be insufficient due to the remote and inaccessible nature of many areas. The integration of new technologies, such as remote sensing, GIS mapping, and environmental DNA sampling, combined with the knowledge of local communities and field biologists, allows for more effective disease tracking in wildlife populations. By employing a range of tools and techniques, researchers can identify disease outbreaks earlier, trace transmission pathways, and better predict how diseases may spread across species barriers. This proactive approach is vital for preventing zoonotic diseases from becoming public health crises [41,51,83].

Moreover, collaboration across disciplines encourages the sharing of data and resources that can be leveraged for both research and policymaking. In the context of the Amazon Biome, international partnerships between academic institutions, government agencies, and non-governmental organizations are instrumental in addressing the region’s unique challenges. For instance, initiatives aimed at preserving biodiversity and monitoring environmental health can also provide critical insights into the spread of infectious diseases. Similarly, wildlife conservation efforts that incorporate veterinary health assessments can help identify pathogens that may threaten not only animal populations but also humans and domestic livestock. The One Health approach fosters an environment in which these intersecting interests can be addressed simultaneously, leading to more sustainable solutions [1,2,41,79].

In addition to facilitating research and surveillance, interdisciplinary collaboration within the One Health framework promotes more effective policymaking. Policymakers need to rely on scientific expertise from various fields to develop strategies that address the root causes of disease emergence, such as habitat destruction, climate change, and human encroachment into wildlife habitats. By working alongside scientists and health professionals, decision-makers can implement policies that protect both public health and biodiversity. In the Amazon, this might involve stricter regulations on land use, efforts to reduce deforestation, or enhanced conservation programs that prioritize ecosystem health. Collaborative policymaking ensures that health interventions are not only reactive but also preventative, addressing the environmental and social factors that contribute to disease outbreaks in the first place [5,7,83].

Finally, interdisciplinary collaboration within the One Health framework empowers local communities, who are often the first to encounter wildlife and notice changes in the environment. In the Amazon Biome, Indigenous peoples and local populations possess invaluable knowledge of the ecosystems they inhabit. Collaborating with these communities and integrating their traditional ecological knowledge into modern scientific practices can lead to more nuanced and culturally appropriate health interventions. This collaboration also fosters trust between researchers and local populations, ensuring that disease surveillance, prevention, and control efforts are more sustainable and better suited to the needs of the region. By bringing together a wide range of disciplines and perspectives, the One Health approach not only strengthens disease control efforts but also promotes a more inclusive and equitable form of global health research [2,3,4,5].

## 5. Discussion

The intersection of wildlife, infectious and parasitic diseases, and human activity in the Amazon Biome is increasingly recognized as a critical area of study, particularly within the framework of One Health [22]. Previous research has established that the Amazon’s biodiversity serves as a natural reservoir for many pathogens that have the potential to spill over into domestic animals and human populations [17]. However, our understanding of the prevalence and impact of these diseases remains limited, largely due to the challenges of conducting comprehensive surveillance in such a remote and complex environment [51]. Studies like those on *Leishmania* spp., *Trypanosoma* spp., and various parasitic helminths have illustrated the significant role of wild animals as hosts for zoonotic agents, but much remains to be explored regarding the broader spectrum of infectious agents and their ecological dynamics in this biome [61,75].

The encroachment of human activity into the Amazon, including deforestation, agriculture, and urbanization, has exacerbated the risk of disease transmission. Habitat destruction and fragmentation increase contact between wildlife, livestock, and human populations, facilitating the emergence of zoonotic diseases [12]. For example, studies on diseases like yellow fever and leptospirosis in the Amazon have demonstrated that land use changes can shift disease vectors and reservoirs, increasing the risk of outbreaks [24]. The implications of these findings suggest that conserving natural habitats not only protects biodiversity but also reduces the likelihood of zoonotic spillovers, reinforcing the One Health principle that ecosystem health is integral to human and animal health [7,41].

In terms of parasitic diseases, research has shown that wild animals in the Amazon Biome harbor a wide range of parasites, many of which have zoonotic potential. Helminthic infections, protozoan parasites such as *Toxoplasma gondii*, and various ectoparasites have been documented in species ranging from primates to birds [64,68,75]. The role of these animals as reservoirs of parasites that can infect humans and domestic animals is a growing concern. For instance, the presence of Toxoplasma in Amazonian wildlife poses a risk to local communities and livestock [86]. This underlines the importance of integrated monitoring programs that include wildlife, humans, and domestic animals, as parasitic diseases often have complex transmission cycles that involve multiple hosts and vectors [81,83].

The role of climate change as a driving factor for disease dynamics in the Amazon cannot be overlooked [13]. Shifts in temperature and precipitation patterns are altering the habitats and behaviors of both wildlife and disease vectors. Studies have predicted that, as the Amazon’s climate changes, diseases such as malaria and dengue, which are transmitted by mosquitoes, could see shifts in their geographic distribution [16]. Similarly, climate change may affect the life cycles of parasites and their intermediate hosts, leading to changes in infection patterns. This further complicates disease management and highlights the need for long-term studies that track the impact of climate change on disease transmission within this unique ecosystem [76,85].

Another critical aspect that has emerged from the literature is the underrepresentation of comprehensive disease surveillance in wild animals in the Amazon. While research has been conducted on certain zoonotic diseases, such as rabies [39,40,41] and hantavirus [46,47], there is a notable gap in the systematic collection of data on less well-known pathogens. Future research should prioritize the establishment of large-scale surveillance networks that can detect the early signs of disease emergence [41,51]. These networks would be most effective if they combined veterinary, medical, and ecological expertise, ensuring that all relevant aspects of disease transmission are considered in a holistic manner.

The lack of resources and infrastructure in many parts of the Amazon also poses a significant challenge to conducting research and implementing disease control measures. Improving the local capacity for disease monitoring and intervention is essential for mitigating the risks associated with infectious and parasitic diseases [5,7,13]. Collaborative efforts between governments, international organizations, and research institutions will be necessary to develop sustainable solutions. This might include the training of local professionals in wildlife health monitoring and the establishment of diagnostic laboratories equipped to handle a range of pathogens. The One Health approach provides an ideal framework for addressing the complex interactions between wildlife, humans, and the environment in this biodiversity hotspot [5,37,79,85]. Future research should focus not only on identifying and characterizing new pathogens but also on understanding the ecological and social factors that drive disease emergence. By doing so, we can develop more effective strategies for preventing zoonotic spillovers and protecting the health of both human and animal populations in the Amazon.

## 6. Conclusions

The Amazon Biome, one of the most biodiverse ecosystems on Earth, is a critical region for understanding the interplay between infectious and parasitic diseases, wildlife, domestic animals, and human populations. Studying these diseases in wild animals is essential not only for biodiversity conservation but also for public health, given the heightened risk of zoonotic spillovers as human activities increasingly disrupt natural habitats.

Adopting a One Health approach, which integrates animal, human, and environmental health, is crucial for effective disease surveillance, early outbreak detection, and prevention strategies. Such an interdisciplinary framework promotes collaboration across ecological, veterinary, and public health fields, ensuring the comprehensive management of health risks in the Amazon.

Future research should focus on identifying key reservoirs and vectors among Amazonian wildlife and understanding the ecological and anthropogenic factors driving disease transmission. Increased investment in field studies, diagnostic tools, and monitoring programs is vital for advancing knowledge in this area. Protecting the health of Amazonian wildlife not only safeguards global public health but also preserves the integrity of one of the planet’s most vital ecosystems.

## Data Availability

No new data were created or analyzed in this study. Data sharing is not applicable to this article.
